# New *Bacillus subtilis* Strains Isolated from *Prosopis glandulosa* Rhizosphere for Suppressing *Fusarium* Spp. and Enhancing Growth of *Gossypium hirsutum* L.

**DOI:** 10.3390/biology12010073

**Published:** 2022-12-31

**Authors:** Ali Abdelmoteleb, Lizbeth Moreno-Ramírez, Benjamín Valdez-Salas, Mahmoud F. Seleiman, Salah El-Hendawy, Khalid J. Aldhuwaib, Majed Alotaibi, Daniel González-Mendoza

**Affiliations:** 1 Botany Department, Faculty of Agriculture, Menoufia University, Shebin El-Kom 32511, Egypt; 2 Instituto de Ciencias Agrícolas, Universidad Autónoma de Baja California (ICA-UABC), Carretera a Delta s/n C.P. 21705, Ejido Nuevo León, Mexicali 21100, BC, Mexico; 3 Instituto de Ingeniería, Universidad Autónoma de Baja California, Calle de la Normal S/N y Boulevard Benito Juárez, Mexicali 21100, BC, Mexico; 4 Plant Production Department, College of Food and Agriculture Sciences, King Saud University, P.O. Box 2460, Riyadh 11451, Saudi Arabia; 5 Department of Crop Sciences, Faculty of Agriculture, Menoufia University, Shebin El-Kom 32514, Egypt; 6 Department of Agronomy, Faculty of Agriculture, Suez Canal University, Ismailia 41522, Egypt; 7 School of Biological Sciences, University of Reading, Reading RG6 6EX, UK

**Keywords:** PGPR, AFM, lipopeptides, *Bacillus subtilis*, IAA, gibberellin, cotton plants

## Abstract

**Simple Summary:**

*Fusarium* species can cause serious damage to agricultural crops. Due to the significant losses in crop production along with the harmful effects of the chemical control of plant diseases on human health and the environment, the use of biocontrol agents for the efficient control of *Fusarium* spp. is becoming an important issue. In the present study, three *bacillus subtilis* strains (LDA-1, LDA-2, and LDA-3) were examined for their potential to promote cotton growth and act as biocontrol agents against *Fusarium* spp. All Bacillus strains exhibited defensive effects in cotton plants against phytopathogenic *Fusarium* spp. The results suggest that the antagonism mechanism of *Bacillus* strains against phytopathogenic *Fusarium* spp. could be due to the ability of bacterial strains to produce lipopeptides and other molecules with antifungal activities. In conclusion, these *Bacillus subtilis* strains can be promised as biocontrol agents, especially in organic and sustainable agricultural systems, and can reduce the extensive use of toxic chemical pesticides in agricultural system.

**Abstract:**

Rhizobacteria from desert plants can alleviate biotic stress and suppress plant diseases, and consequently can enhance plant growth. Therefore, the current study was performed to isolate and identify *Prosopis glandulosa*-associating rhizobacteria based on their antagonistic activity against *Fusarium* species and plant growth-promoting properties. Three bacterial isolates were identified as *Bacillus subtilis*: LDA-1, LDA-2, and LDA-3. The molecular analysis suggests the biosynthesis of the bacteriocins subtilisin and subtilosin, as well as the lipopeptide iturin, by these strains. In addition, the antagonistic study by dual-culture assay showed a high efficacy of all *B. subtilis* strains against phytopathogenic fungi (*Fusarium nygamai*, *F. equisseti*, *F. solani*, *F. solani* ICADL1, and *F. oxysporum* ICADL2) with inhibition percentages ranging from 43.3 to 83.5% in comparison to the control. Moreover, atomic force microscopy (AFM) analysis showed significant differences in the cell wall topography of the *F. solani* ICADL1 among the treated mycelia and untreated control. As a result, these three *B. subtilis* strains were used as bioinoculants for cotton seedlings infected by *F. solani* ICADL1 in pot trials, and the results revealed that the bacterial inoculations as an individual or combined with *F. solani* ICADL1 significantly improved cotton root and stem length, lateral roots, indole acetic acid (IAA), and gibberellic acid (GA_3_) contents, as well as increased antioxidants, flavonoids, and phenols in comparison to those obtained from healthy and infected control plants. In conclusion, the three bacterial strains of *B. subtilis* (i.e., LDA-1, LDA-2, and LDA-3) are considered promising tools as biocontrol agents for *F. solani* and cotton growth promoters, and consequently can be used as bio-ertilizer in sustainable agriculture systems.

## 1. Introduction

Cotton is a globally grown as an annual plant and is one of the most widely utilized fibers in the textile industry, since it can provide about 35% of total fiber worldwide [1]. Its seeds contain a high percentage of oil. Moreover, cotton seeds can be used for human consumption after refining, while seed bark can be utilized to feed animals. In addition, the linter, consisting of short fiber layers adhering to cotton seeds, is applicable in various industries [2]. However, the presence of fungal pathogens during the development of cotton plants is a big threat, because these pathogens can affect the quality and quantity of cotton production [3]. *Fusarium* wilt caused by *Fusarium solani* is an significant disease and is considered one of the most causes of cotton economic losses worldwide [1].

*Fusarium* spp. can cause several diseases, including wilt, seedling disease complex, root rot, and boll rot [4]. It can attack plants through wounds, seeds, and roots, particularly if the border cells of the root cap are damaged [5]. According to diverse reports, the control of *Fusarium* wilt is a challenge, and novel controls as well as alternative options are highly recommended. For example, the use of resistant cultivars, pathogen-free cotton seeds, agrochemicals (e.g., fungicides), or biological antagonists are important to control such fungal pathogens. Although, the use of chemical fungicides can successfully inhibit pathogen growth and reduce plant diseases, they can have harmful impacts on plants, human health, and some beneficial microorganisms. In addition, the application of chemicals can cause a risk to sustainable agriculture as a result of their accumulation in water and soil [6]. Additionally, the overuse of chemical fungicides can expose phytopathogens to sublethal amounts of the fungicide on a continuous basis, which can result in an emergence for fungicide resistance [7].

Hence, new scientific innovations on the use of beneficial microorganisms to prevent plant disease and improve crop productivity are crucial given the rise in demand for safe, chemical-free production methods [8]. In this sense, the use of beneficial microbes from a native area (i.e., autochthonous microorganisms) is an eco-friendly alternative to agrochemicals. Beneficial microbes are characterized by their ability to produce several antibacterial and antifungal antibiotics, which can effectively suppress phytopathogens under field conditions by several mechanisms such as antibiosis, parasitism, competition, lytic enzymes, and induced systemic resistance [9,10].

One of the biological control alternatives is the exploitation of plant growth-promoting bacteria (PGPB) with antagonistic activity in the control of plant pathogenic fungi. Among PGPB, *Bacillus* species are some of the most significant biocontrol agents; for example, *Bacillus velezensis* controls plant diseases through inhibition of fungal growth using antibiotic compounds such as cyclic tetrapeptides, direct competition, and the release of hydrolytic enzymes such as β-1,3-glucanases and chitinases [7]. Numerous strains belonging to the genus *Bacillus*, particularly *B. subtilis*, have been shown to be effective and potent in biologically controlling several plant diseases [9]. The genetic composition of *Bacillus* indicates the capacity of antagonistic bacteria to release antimicrobial substances of both a volatile and nonvolatile type, contributing towards reducing plant diseases [11]. Several *Bacillus* strains, including *B. amyloliquefaciens*, *B. mojavensis*, and *B. velezensis*, produce antimicrobial substances such as fengycin (Fen D), iturin (Itu C), bacillomycin (Bmy A), and nonpeptidic antimicrobial substances such as phospholipids, polyketides, and aminosugars that have the ability to suppress the growth of *Fusarium* species [11,12,13]. Additionally, substances such as surfactin, fengycin, and other antimicrobial peptides increase the permeability of the hyphal cells’ plasma membrane, inhibiting their growth. Furthermore, several *Bacillus* species have the ability to release volatiles that induce the deterioration of *F. oxysporum* hypha [13]. Multiple lipopeptides are produced by *Bacillus*, which broadens and improves its range of antifungal and antibacterial activity, as well as facilitating the colonization of biological niches [10].

The biotic stress in plants caused by pathogen attacks leads to harmful reactive oxygen species (ROS) generation and damages the reaction chain, where their excessive synthesis obstructs the normal growth of plants [14]. The potential of plants’ recovery from oxidative stress is based on the detoxification of free radicals to protect vital cellular functions. Beneficial microorganisms can induce the production of defense metabolites such as antioxidants, flavonoids, and phenolic compounds in plants [14,15]. In this context, the severity of wilt in tomato plants inoculated with the biocontrol agent Trichoderma was reduced as a result of the phenolic content increase in plants due to their antimicrobial properties [15]. Moreover, phenolic compounds act as lignin precursors, therefore promoting the lignification of the plant cell wall, which represents a physical barrier to protect plants against the invasion of pathogens [15,16].

Autochthonous microorganisms can be free-living beneficial bacteria, called plant growth-promoting rhizobacteria (PGPR), comprising of different genera such as *Azospirillum*, *Azotobacter*, *Burkholderia*, *Bacillus*, and others [17]. In this respect, *Bacillus* species are considered as biocontrol agents that are nonpathogenic, live in soil associated with plant roots, and produce a broad spectrum of biologically active molecules (e.g., lipopetide antibiotics) for suppressing phytopathogens and plant growth promotion under field or controlled conditions [14,18,19]. Honey mesquite (*Prosopis glandulosa*) is a species of genus *Prosopis*, and these plants are found in arid and semiarid zone ecosystems in the north of Mexico and south of California, USA. This species is characteristic of the vegetation desert ecosystem in Baja California, Mexico [20]. The scientific focus on *P. grandulosa* trees is to evaluate their physiological and ecological adaptations to a hyperarid environment. However, the studies about the diversity of *Bacillus* spp. isolated from the rhizosphere of *P. glandulosa* and their ability to simultaneously act as a biocontrol agent or biostimulator are limited.

Therefore, the aims of the present study were to (a) characterize and identify three new antagonistic *Bacillus subtilis* strains isolated from the rhizosphere of *Prosopis glandulosa*, (b) to evaluate their ability in vitro to inhibit five *Fusarium* species that can cause root rot in strategies crops, (c) investigate the potential use of *B. subtilis* strains as bioinoculants to enhance and protect cotton seedlings against *Fusarium solani*, and (d) analyze the biochemical changes in the defence-related enzyme activity (antioxidants, total phenols, and flavonoids) of cotton plants grown under different treatments. Our hypothesis was that the three new antagonistic *Bacillus subtilis* strains isolated from the rhizosphere of *Prosopis glandulosa* could inhibit *Fusarium* species that can cause root rot in strategic crops.

## 2. Materials and Methods

### 2.1. Isolation of Antagonistic Bacillus *spp.* from Honey Mesquite Rhizosphere

*Bacillus* spp. used in the current study was isolated from the rhizospheric soil of honey mesquite (*Prosopis glandulosa*) trees located in an uncultivated zone in the Mexicali Valley (32°24’34 N/115°11’16 W), Mexico. Soil samples were collected in triplicate at depths of 10–15 cm in sterile polyethylene bags using a sterile spatula. Immediately, the soil samples were transferred into the Laboratory of Biotechnology, ICA-UABC, Mexico, for bacterial isolation. Soil samples were passed through sieve and then mixed and homogenized. To obtain 10^−1^ dilution, 10 g of homogenized soil was suspended in 90 mL distilled water and shaken for 15 min at 150 rpm. Serial dilution was performed up to 10^−6^; 100 µL of each dilution was spread on plates of nutrient agar and incubated at 37 °C for 24 h. Single colonies were selected and purified by streaking on nutrient agar plates. Three pure cultures were maintained at 4 °C on nutrient agar slants for further studies.

### 2.2. Isolation of Pathogenic Fungi

Pathogenic fungi were isolated from infected alfalfa (*Medicago sativa* L.) and cotton plants with root rot symptoms collected from cultivated fields in Mexicali Valley, Mexico. The infected alfalfa and cotton roots were cut into small pieces and surface-sterilized by NaOCl 0.5% for 3 min. Then, the small pieces of roots were rinsed three times using distilled water and were cultivated on potato dextrose agar (PDA). After 4 days at 25 °C, fungal growth was subcultured by hyphal tip transfer technique on PDA plates, and incubated at 25 °C. Two pure cultures of pathogenic fungi alfalfa and cotton plant were obtained and stored in slant agar at 4 °C for further investigation.

### 2.3. Molecular Identification of Bacterial Isolates

A single colony of each isolate was inoculated in a 250 mL Erlenmeyer flask containing 100 mL of nutrient broth media, and incubated for 24 h at 37 °C. Then, 1 mL of each culture was centrifuged at 10,000 rpm for 6 min at 4 °C, and the pellet was used for DNA extraction by phenol-chloroform method, and DNA was stored at −20 °C until used [21]. 16S rDNA sequence of each isolate was amplified using universal primers 27F and 1495R (Table 1). PCR reaction mixture (30 µL) consisted of 2 µL DNA, 3 µL t PCR(Taq) buffer (10x), 1µL 10 mM dNTP, 2.5 µL 50 mM MgCl_2_, 0.3 µL (10 mM) of each primer 27F and 1495R [22], and 0.3 µL Taq polymerase (5 units/µL). PCR reaction was run in PCR thermal cycler (BIO-RAD) using the thermal profile as follows: 4 min at 94 °C for the initial denaturation, and then 35 cycles for 1 min at 94 °C, 1 min at 59 °C, 2 min at 72 °C, and final cycle for 7 min at 72 °C. PCR products were visualized by gel electrophoresis, purified by “Wizard^®^ SV Gel and PCR Clean-Up System” kit (Promega-A9281), and were sent to GENEWIZ^®^ Lab (South Plainfield, NJ, USA) for sequencing. Using BLAST search in NCBI GenBank (http://www.ncbi.nlm.nih.gov/ (Accessed on 1 November 2022)), 16S rDNA sequences were compared with the most similar species. The construction of the neighbor-joining phylogenetic tree was based on 16S rDNA sequences using MEGA10.1.5 software.

### 2.4. Amplification of Lipopeptide Genes

Lipopeptide genes (subtilisin, subtilosin, and iturin) were detected in three *Bacillus* isolates by PCR amplification technique using extracted genomic DNA of each strain and specific primers [24], listed in Table 1. PCR amplification was performed as follows: initial denaturation at 94 °C for 4 min, 30 cycles of (denaturation 30s at 94 °C, annealing 30s at 58 °C, and extension 1 min at 72 °C) and final extension was 7 min at 72 °C. The PCR results were confirmed by running in 1.5% agarose gel electrophoresis at 90 V for 40 min, and then visualized under UV light and documented using the Multidoc-It Digital Imaging system (UVP, Upland, CA, USA).

### 2.5. Molecular Identification of Pathogenic Fungi

Fungal isolates were cultured in PDA at 25 °C for 7 days, and approximately 200 mg of mycelium were used for genomic DNA extraction by phenol/ chloroform method [25]. The ribosomal rRNA gene of isolated fungi was amplified by the universal primers internal transcribed spacer ITS4 and ITS5, as shown in Table 1 [23]. The reaction mixture (30 µL) contained 1 µL of cDNA, 2 µL of each primer (ITS4 and ITS5), 3 µL of Buffer (10×), 2.5 µL of MgCl_2_, 1 µL of dNTPs, and 0.3 µL of Taq polymerase enzyme. PCR reaction was performed in a thermal cycler (BIO-RAD) as follows: initial denaturation at 95 °C for 3 min, 30 cycles (95 °C for 30 s, annealing 55.5 °C for 30 s, and extension at 72 °C for 1 min), followed by a final extension at 72 °C for 7 min. The PCR products were run on a 1% agarose gel containing ethidium bromide and visualized by the Multidoc-It (UVP) digital imaging system. The PCR products were purified by the Purelink^®^ PCR Purification kit (Invitrogen, Waltham, MA, USA) and sent to Genewiz Lab, New Jersey, USA for sequencing and subsequent computer analysis. The sequences obtained were analyzed by BLAST search on the website of the National Center of Biotechnology Information (NCBI) (http://www.ncbi.nlm.nih.gov/ (Accessed on 1.November.2022)), to detect the identical species in the database of GenBank^®^. The phylogenic tree was constructed based on ITS sequences via neighbor-joining analysis using MEGA10.1.5 software. The obtained sequences were aligned together with homologous sequences obtained from the GenBank database.

### 2.6. Indole Acetic Acid (IAA) Production by Bacterial Isolates

The bacterial isolates were assessed for IAA production in Luria Bertani (LB) medium, amended with 0.5 mg/ mL L-tryptophan. Erlenmeyer flasks (250 mL) containing 50 mL of LB medium were inoculated by 1 mL overnight-grown cultures (10^8^ CFU) of the isolates and incubated in dark with shaking at 120 rpm for 5 days at 35 ± 2 °C. After incubation, the cultures were centrifuged at 10,000 rpm for 12 min at 4 °C; 1 mL of supernatant was mixed with 2 mL of Salkowski’s reagent, and then incubated for 30 min in the darkness at room temperature. The development of the pink color was considered evidence of IAA production [26]. For quantitative determination of IAA produced, the absorbance was measured at 530 nm, and the optical density was compared to the standard curve of IAA.

### 2.7. Gibberellic Acid (GA3) Production by Bacterial Isolates

The gibberellin assay was performed using the colorimetric method as described by Abdelmoteleb et al. [27]. Bacterial isolates were inoculated in a nutrient broth medium and incubated at 35 ± 2 °C on a rotary shaker for 3 days. The culture was centrifuged for 5 min at 10,000 rpm to collect the bacterial cells. For estimating the gibberellic acid production, 15 mL of culture supernatant was transferred into test tubes and then mixed with 2 mL of zinc acetate solution. After 2 min at room temperature, 2 mL of potassium ferrocyanide (10.6%) was added to the mixture, then centrifuged for 8 min at 4000 rpm. A total of 5 mL of supernatant was mixed with an equal volume of 30% hydrochloric acid and incubated for 75 min at 27 °C. The optical density was measured by a UV-Vis spectrophotometer at 254 nm, and 5% hydrochloric acid served as a blank. Gibberellin concentrations were calculated by comparing the readings with the standard curve of gibberellic acid (100–1000 μg/mL).

### 2.8. Antifungal Activity of Bacterial Isolates in Dual-Plate Assay

Antifungal activity of bacterial isolates against phytopathogenic fungi (*Fusarium nygamai*, *F. equisseti*, *F. solani*, *F. solani* ICADL1, and *F. oxysporum* ICADL2) was performed using dual-culture technique [28]. Mycelial disc (0.6 cm) from the old culture of each pathogenic fungus was transferred into PDA plate center. Each bacterial isolate was inoculated in a square pattern around the pathogenic disc, where the distance between each side of the square and the plate center was about 2 cm. PDA plates inoculated by fungal discs alone served as controls. All treatments were conducted in triplicate. After 7 days of incubation at 26 ± 2 °C, the diameter of fungal colonies was measured. Then, the inhibition percentage of radial growth was calculated using the following equation:

$$I\%=\frac{C-T}{C}\times 100$$

where I = inhibition percentage of the radial growth, C = colony diameter of control, and T = fungal colony diameter of dual culture plates.

### 2.9. Morphological Analysis of Phytopathogen in Contact with Antagonistic Bacteria Using Atomic Force Microscopy (AFM)

Atomic force microscopy (AFM) analysis was performed at the Institute of Engineering, UABC-Mexicali, Mexico, to observe morphological changes in the phytopathogen after being exposed to direct contact with antagonistic bacteria. Using a wet chamber designed to observe fungal morphology by AFM, both bacteria and phytopathogenic fungi were grown in contact for 5 days in special slides. After that, an aliquot was observed in AFM to be able to evaluate the effect on the morphology of the phytopathogenic fungus [29].

### 2.10. Effect of Antagonistic Bacteria on Cotton Seedlings Exposed to Phytopathogen F. solani ICADL1

The ability of antagonistic bacteria to reduce the damage caused by *F. solani* ICADL1 was investigated using inoculated cotton seeds with antagonistic bacteria or uninoculated ones (control). Then, seeds were planted in pots containing sterile peat moss. After 14 days, conidial suspension of *F. solani* ICADL1 was added. The 10 treatments (Table 2) were arranged in completely randomized design with 3 biological replications, and 10 seeds were sown in each replicate. After 28 days of sowing, the physiological parameters, including root and stem length and number of secondary roots, were measured. Moreover, biochemical traits such as IAA and GA_3_ were analyzed as described by Akladious et al. [6], while antioxidants, flavonoids, and phenols were analyzed as described by Aryal et al. and Ruiz-Cisneros et al. [30,31].

### 2.11. Statistical Analyses

Statistical analyses of all data were performed by one-way analysis of variance (ANOVA) using PASW statistics 21.0 (IBM Inc., Chicago, IL, USA). The means of different treatments for each trait were compared using Duncan’s multiple range test at *p* ≤ 0.05. All means of different treatments for each trait were acquired from the raw data of three replications and expressed as mean ± standard error (SE).

## 3. Results

**Figure 1:**
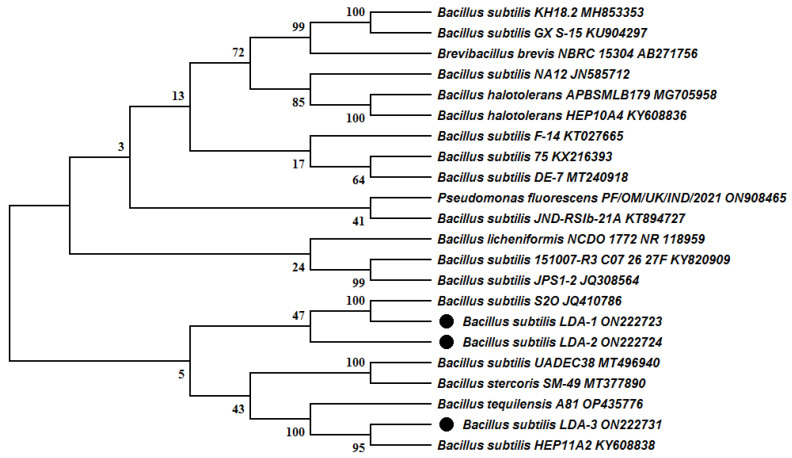
Neighbor-joining phylogenetic tree (values at branching points indicate bootstrap) based on 16S rDNA sequences of isolated *Bacillus subtilis* strains LDA-1, LDA-2, and LDA-3. (bootstrap values expressed as percentages of 1000 replications).

In the present study, three bacterial strains were obtained from the rhizosphere of *Prosopis glandulosa*, and the molecular analysis and BLAST search using 16S rRNA sequence revealed that they belong to the genus of *Bacillus*. Furthermore, the comparison of 16S rRNA sequences of the three strains showed high similarity to *Bacillus subtilis*. Therefore, the three strains were named and submitted to the GenBank as follows: *Bacillus subtilis* strain LDA-1 (ON222723), *Bacillus subtilis* strain LDA-2 (ON222724), and *Bacillus subtilis* strain LDA-3 (ON222731). The analysis of 16S rRNA sequences showed that the isolated strains LDA-1 and LDA-2 lie on the same phylogenetic tree branch close to *Bacillus subtilis* strain S2O (JQ410786), while strain LDA-3 lies on the other phylogenetic tree branch close to *Bacillus subtilis* strain HEP11A2 (KY608838) and *Bacillus tequilensis* strain A81 (OP435776) (Figure 1).

**Figure 2:**
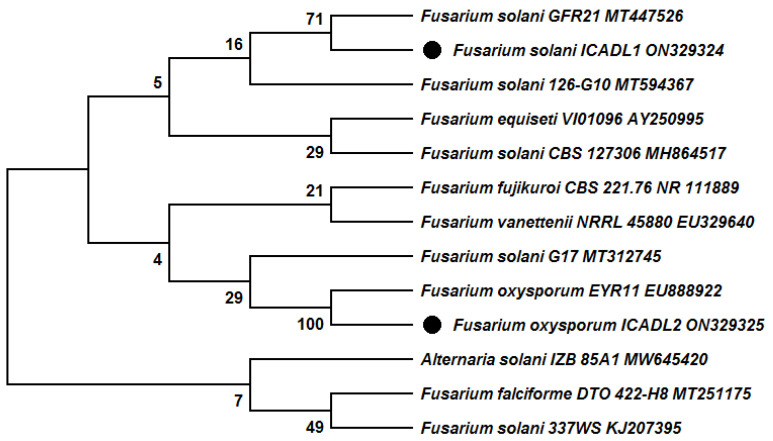
Phylogenetic tree constructed by neighbor-joining method (values at branching points indicate bootstrap) based on ITS sequences showing the relationship of isolated *Fusarium* strains to closely related species. (bootstrap values expressed as percentages of 1000 replications).

Two phytopathogen fungal strains were isolated from alfalfa and cotton roots and identified based on ITS sequences. The amplification of the ITS region was successfully performed and the sequence data were used for the BLAST search in the NCBI database. The BLAST search revealed that the two isolated fungal strains are members of the genus *Fusarium*. The ITS sequences were aligned with the available sequences in the NCBI database to construct the phylogenetic tree based on ITS sequences by the neighbor-joining (N-J) method. The phylogenetic tree revealed that the first strain exhibited similarity (71%) to *Fusarium solani* strain GFR21(MT447526), while the second strain was most relative to *Fusarium oxysporum* strain EYR11 (EU888922) with 100% similarity (Figure 2). Therefore, the two isolated fungal strains were identified and recorded in GenBank as *Fusarium solani* strain ICADL1 (ON329324) and *Fusarium oxysporum* strain ICADL2 (ON329325), respectively.

For the detection of genes encoding antimicrobial lipopeptides (subtilosin, subtilisin, and iturin) from *Bacillus subtilis* strains, DNA samples extracted from isolated *B. subtilis* strains (LDA-1, LDA-2, and LDA-3) were used through PCR reaction using specific primers for each gene (Table 1). PCR results revealed amplification of both subtilosin and subtilisin in all strains (Figure S1). Meanwhile, the iturin-encoding gene was amplified in two strains, *B. subtilis* LDA-1 and LDA-3, while the other strain, *B. subtilis* LDA-2, exhibited no indication of the presence of the iturin gene. Each one of the lipopeptides subtilosin, subtilisin, and iturin’s genes exhibited one specific visible band around 334, 704, and 885 bp in length, respectively.

**Figure 3:**
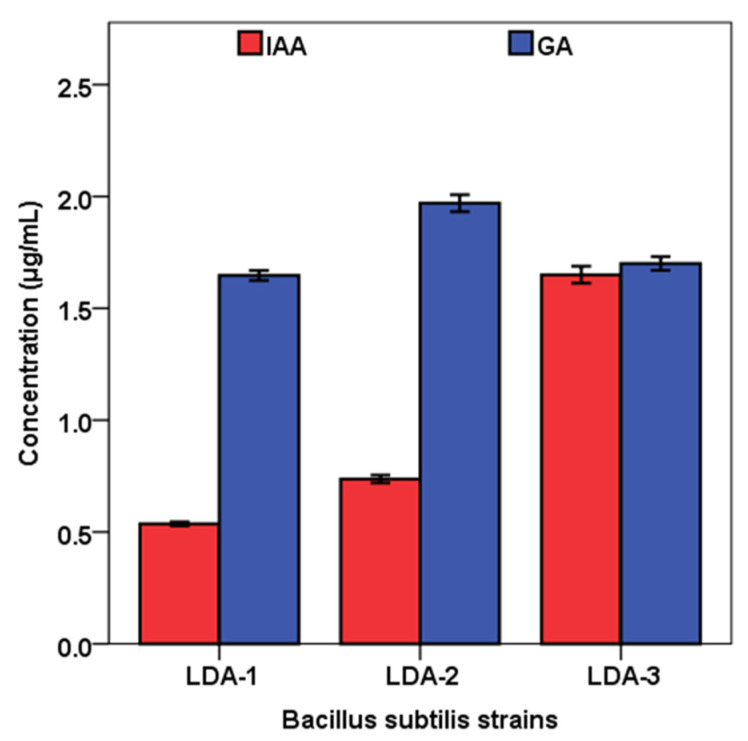
Indole acetic acid (IAA) and gibberellic acid (GA_3_) produced by *Bacillus subtilis* strains LDA-1, LDA-2, and LDA-3. Values = mean ± SE, (n = 3).

On the other hand, three *B. subtilis* strains were able to produce IAA and GA in liquid culture in the range of 0.53−1.65 μg/mL and 1.64−1.97 μg/mL, respectively (Figure 3). The highest IAA content (1.65 ± 0.04 μg/mL) was recorded by *B. subtilis* strain LDA-3 followed by strains LDA-2 (0.74 ± 0.02 μg/mL) and LDA-1 (53 ± 0.01 μg/mL), respectively, while the highest concentration of GA was recorded by *B. subtilis* strain LDA-2 (1.97 ± 0.04 μg/mL) compared to that obtained with LDA-1 (1.64 ± 0.02 μg/mL) or with LDA-3 (1.7 ± 0.03 μg/mL).

**Figure 4:**
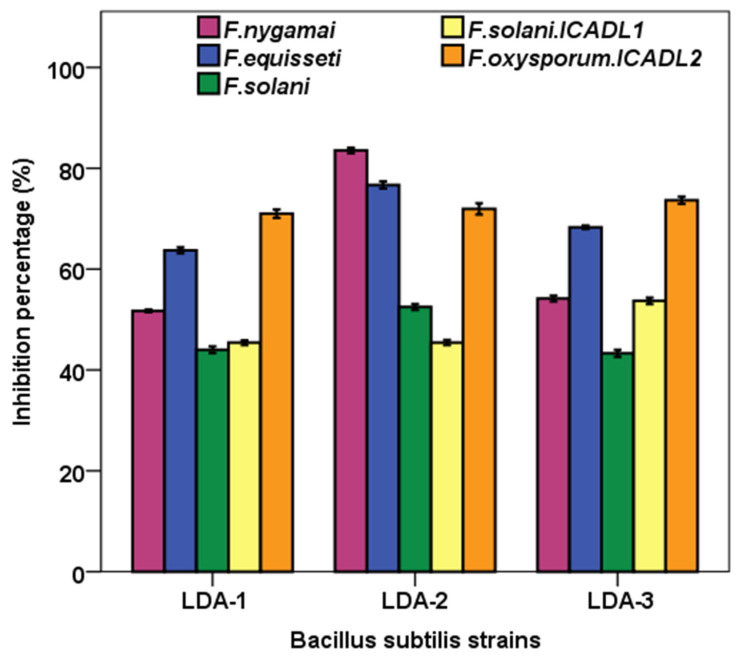
Growth inhibition of phytopathogenic fungi by *B. subtilis* strains isolated from the rhizosphere of *P. glandulosa* plants, in dual-plate assay. Values = mean ± SE, (n = 3).

The three bacterial strains (LDA-1, LDA-2, and LDA-3) significantly (*p* < 0.05) suppressed the radial fungal growth of *Fusarium* strains, namely *F. nygamai*, *F. equisseti*, *F. solani*, *F. solani* ICADL1, and *F. oxysporum* ICADL2 in dual cultures compared to controls without bacteria after 6 days of incubation (Figure 4). The inhibitory effect of antagonistic bacterial strains against fungi was varied from 43.3 to 83.5% growth inhibition. The means comparison of the inhibitory effect of all bacterial strains on the mycelial growth of each fungus revealed that the maximum inhibition (83.5 ± 0.53%) was obtained with *B. subtilis* LDA-2 against *F. nygamai*. In contrast, the lowest inhibition percent (43.3% ± 0.69) was observed on *F. solani* when treated with *B. subtilis* LDA-3.

In the case of *F. nygamai*, *F. equisseti*, and *F. solani*, the *B. subtilis* LDA-2 exhibited the highest significant (*p* < 0.01) inhibition rates; while in the case of *F. solani* ICADL1, the highest significant (*p* < 0.01) inhibition rate was recorded by *B. subtilis* LDA-3. All bacterial strains inhibited the growth of *F. oxysporum* ICADL2 by more than 70% compared to the control, but without significant differences among them.

**Figure 5:**
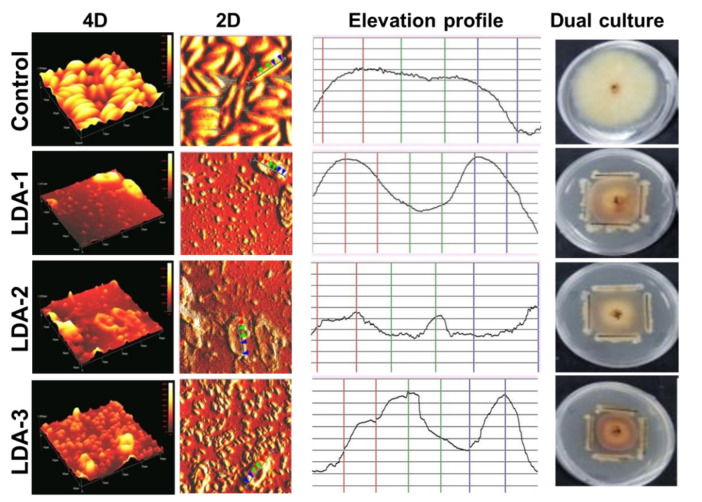
Atomic force microscopy (AFM) images of *F. solani* strain ICADL1 treated by *B. subtilis* strains LDA-1, LDA-2, and LDA-3 illustrating the effect of antagonistic *B. subtilis* strains on cell wall topography of mycelia of *F. solani* ICADL1. The red, green, and blue arrows of the 2D view images correspond to the points outlined in the elevation profile.

Atomic force microscopy (AFM) analysis showed the effect of antagonistic activity of bacterial strains on the cell wall topography of *F. solani* ICADL1, where 4D and 2D images showed significant differences in the length and heights (maximum and minimum) of the fungal cell surface between the mycelia treated with bacteria and the untreated control (Figure 5).

Untreated mycelia showed a regular surface, while mycelia treated with bacterial strains showed a change in the evaluated variables (Table 3). Strain LDA-1 caused a significant (*p* < 0.01) increase in maximum height in comparison to others. In contrast, mycelia treated with strain LDA-3 significantly (*p* < 0.05) decreased in length and height. Thus, it is revealed from AFM results that *B. subtilis* strains LDA-1, LDA-2, and LDA-3 are effective antagonists against *F. solani* ICADL1, therefore altering its cell surface topography.

Finally, the efficacy of three *B. subtilis* strains, LDA1, LDA2 and LDA-3 (individually and mixed) for plant growth promotion, as well as their ability to protect cotton seedlings against *F. solani* ICADL1, had been assessed. The results noted that *B. subtilis* strains stimulated both infected and healthy cotton seedlings’ growth compared to controls. Among all treatments, inoculation of noninfected cotton seedlings with *B. subtilis* LDA-2 significantly (*p* < 0.05) increased stem length. In addition, in healthy plants, two strains (LDA-2 and LDA-3), and in *F. solani*-infected plants, LDA-1, caused a significant (*p* < 0.05) increase in root length compared to healthy and infected controls, respectively. Generally, the effect of bacterial inoculation on root and stem length was not significant in most of treatments. In contrast, all treatments of bacterial inoculation, except *B. subtilis* LDA-1 in infected seedlings (LDA-1 + F), significantly (*p* < 0.05) increased secondary root formation (Table 4). The results of this study demonstrated that *F. solani* infection and *B. subtilis* treatments in cotton seedling therapy altered plant development hormones IAA and GA_3_ (Table 4).

**Figure 6:**
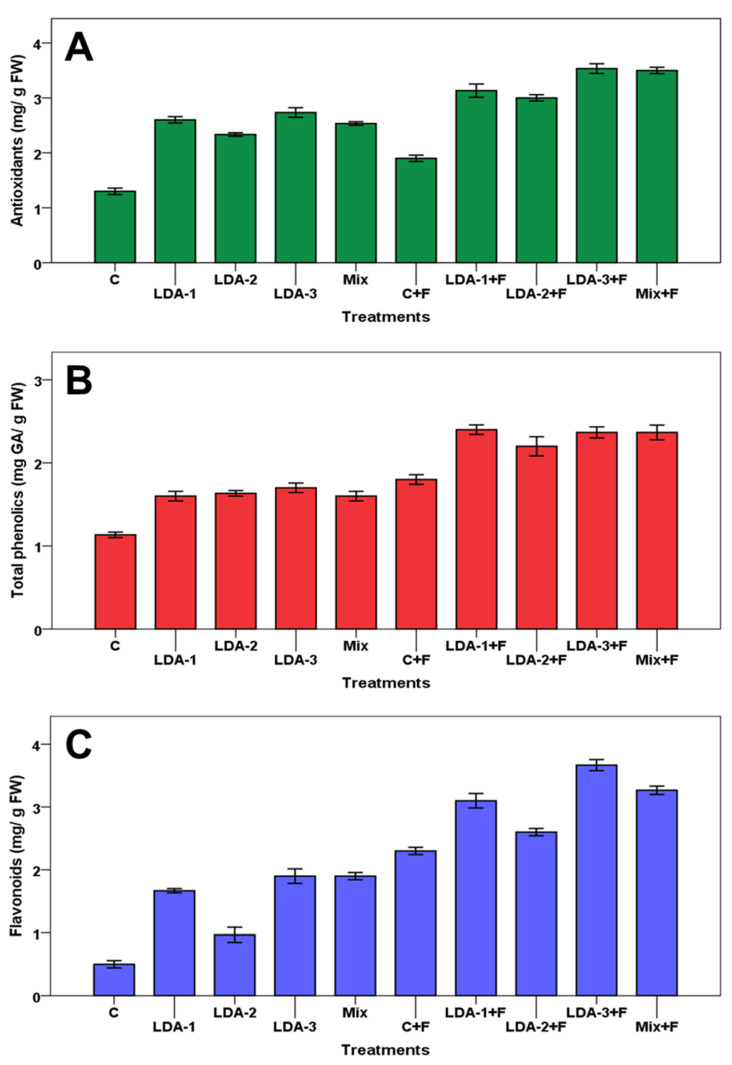
Effect of *B. subtilis* strains (LDA-1, LDA-2, and LDA-3) on antioxidants (**A**), phenolic content (**B**), and flavonoids (**C**) of cotton seedlings infested by *F. solani* ICADL1. C = control, F = *F. solani* ICADL1. The data are presented as means of three replicates, ± standard error (SE).

The inoculation of cotton seedlings with *B. subtilis* increased the auxin and gibberellin production. On the other hand, plants infected with *F. solani* had lower levels of auxin and gibberellins. However, coinoculation of cotton seedlings by *B. subtilis* and *F. solani* increased both IAA and GA_3_ and helped to ameliorate biotic stress. For understanding the role of bacterial strains in improving cotton seedlings’ growth and protecting them against soil-borne *Fusarium solani* wilt disease, the antioxidant, total phenolic content, and flavonoids were evaluated. The results revealed that antioxidant capacity, total phenolic content, and flavonoids were significantly increased in inoculated plants with *B. subtilis* (strains LDA-1, LDA-2, and LDA-3) and *F. solani* ICADL1 (individual or in combination) in comparison to both healthy and diseased controls (Figure 6). In this concern, the antioxidant capacity, total phenolic content, and flavonoids of healthy cotton seedlings treated with separated or combined *B. subtilis* strains increased by up to about 2-, 1.4-, and 3.8-fold, respectively, higher than the untreated healthy control. Similarly, treatments with both the pathogen (*F. solani* ICADL1) + antagonist (*B. subtilis* strains LDA-1, LDA-2, and LDA-3) exhibited the maximum significant increase in antioxidant capacity, total phenolic content, and flavonoids by 2.6-, 2-, and 7.2-fold, respectively, compared to the healthy control. However, treatment with the pathogen alone significantly increased the content of antioxidant capacity, total phenolic content, and flavonoids by up to 1.4-, 1.6-, and 4.6-fold, respectively, when compared with the healthy control.

## 4. Discussion

Rhizospheric soil is considered a valuable source of rhizobacteria that has potential capacities and represents an eco-friendly solution for suppressing soil-borne diseases. The isolation of new strains of antagonistic microorganisms is very important for improving and developing biological control techniques and suppressing phytopathogens. In this study, three *B. subtilis* strains (LDA-1, LDA-2, and LDA-3) isolated from the rhizosphere of honey mesquite (*P. glandulosa)* showed capacity for the biocontrol of *Fusarium* spp., and the stimulation of plant growth. The potential of *Bacillus* species to suppress plant pathogenic fungi can be due to the secretion of antifungal molecules such as lipopeptides and hydrolytic enzymes [8]. In this regard, the dual-culture assay revealed that the three *B. subtilis* strains (LDA-1, LDA-2, and LDA-3) have effective and potent antifungal activity against *F. nygamai*, *F. equisseti*, *F. solani*, *F. solani* ICADL1, and *F. oxysporum* ICADL2. In this context, *B. subtilis* isolate k4-4 and *B. subtilis* isolate GH3-8, obtained from the citrus rhizosphere, suppressed the growth of *F. equiseti*, *F. brachygibbosum*, and *F. oxysporum* by 72.22 and 68.93% mycelial growth inhibition, respectively [32]. Similarly, after 7 days of dual-culture-plate incubation, *B. subtilis* GM2 and *B. subtilis* GM5 showed an inhibitory effect against *Fusarium* sp., *F. avenaceum*, *F. redolens*, *F. oxysporum*, and *F. solani* by inhibition rates ranging from 43.2 to 59.5% and from 54.1 to 66.8%, respectively [8]. In the current study, the inhibition percentage of fungal growth via the isolated *B. subtilis* strains ranged from 43.3 to 83.5%, and this is comparatively higher than the other previously reported strains, as mentioned above. This can be due to the antagonistic properties of *Bacillus* species that can protect plants against phytopathogen attacks through the biosynthesis of different lipopeptides, which can have an inhibitory effect against the phytopathogens [8,17,32]. *Bacillus* species produce multiple antimicrobial compounds that act as biological agents, confer protection, and exert antifungal and/or antibacterial activities against phytopathogens. Such compounds include bacilysin, subtilin, mycobacillin, mycosubtilin, bacillomycin, surfactins, fengycins, and iturins [33]. It was noted that *B. subtilis* CW14 antifungal substances can reduce the mycelium dry weight of *Aspergillus ochraceus*, and suppress mycelial growth and spore formation [34]. *B. velezensis* CE 100 inhibited the mycelial growth of *Macrophomina phaseolina* and *F. oxysporum* f. sp. *fragariae* by 64.7% and 55.2%, respectively, due to its ability to produce chitinases and β-1,3-glucanases that can degrade the fungal cell wall [7].

Therefore, our results suggest that antimicrobial lipopeptide genes found in *B. subtilis* strains (subtilosin, subtilisin and iturin) might be responsible for the inhibition of the pathogen’s growth. In addition, PCR screening showed that the strains LDA-1 and LDA-3 contain three genes encoding for lipopeptides (subtilosin, subtilisin, and iturins). The strain LDA-2 had two genes encoding for lipopeptides (subtilosin and subtilisin). These antimicrobial lipopeptide-encoding genes have been identified in some *Bacillus* species, in particular strains of *B. subtilis* as well as *B. amyloliquefaciens* [8]. Similarly, essential genes of antimicrobial peptide production, including subtilosin and iturin, have been detected in *Bacillus* sp. P5 and *Bacillus* sp. C3 through PCR assays and sequencing [35]. It is important to mention that the simultaneous production of more than one lipopeptide in the current study could be crucial for the efficacy of *B. subtilis* strains against phytopathogens, as stated by Mardanova et al. [8] and Mora et al. [36]. *B. subtilis* creates biofilms on the plant roots, which encourage the synthesis of lipopeptides and increase their antimicrobial activity. Moreover, *Bacillus* species can be used for rhizosphere applications, and also act as endophytic bacteria that protect the host plant from pathogens [37].

According to our findings, the antagonist bacteria might possess biocontrol genes encoding for lipopeptides and bacteriocin production, which could suppress or/and decrease the growth of phytopathogens. Additionally, the AFM examination of *F. solani* strain ICADL1 showed that treatment with *B. subtilis* strains LDA-1, LDA-2, and LDA-3 resulted in topographical changes in mycelial growth. Similarly, the AFM study of the effect of *Bacillus altitudinis* MS16 on *Sclerotinia sclerotiorum* revealed significant differences in the height and surface topology among the treated and untreated mycelia [38]. There are a few studies that involve the evaluation of changes in the process of bacteria–fungus interaction, at the morphological level, specifically in the topography of the structural changes of the cell wall of the pathogenic fungi when interacting with *B. subtilis*.

These changes can exhibit challenges in the formation of fungal cell wall, where *B. velezensis* FKM10 can cause destruction in the cell wall and cell membrane of *F. verticillioides*. Moreover, the cell membrane permeability can be damaged by the lipopeptides that are produced from *Bacillus* strains [39]. Therefore, the prescreening for the presence of lipopeptide-encoding genes may be helpful in the selection of strains that have the potential to be successful in the biocontrol of phytopathogens. In the current study, *B. subtilis* strains enhanced cotton seedlings’ growth and significantly increased stem and root length and lateral roots. This could be due to cell division and its elongation. In addition, it can be attributed to the ability of *B. subtilis* strains for synthesizing plant hormones such as IAA and GA, especially IAA, which can stimulate plant growth through promoting lateral root formation and increasing cell volume [40,41]. In another study, the root and shoot growth of wheat seedlings exposed to *F. oxysporum* were stimulated by *B. subtilis* GM2 and were protected against *F. oxysporum* by *B. subtilis* GM5 [8]. On contrary, our results showed that *B. subtilis* strains (individually or mixed) were able to control of the *F. solani* ICADL1 and stimulate the growth of cotton seedlings simultaneously. Thus, the potential of these strains supports their candidacy as strong biocontrol agents and plant growth promoters. *B. subtilis* possesses the ability to directly enhance plant growth by multiple mechanisms, including biofilm formation; nitrogen fixation; the production of phytohormones and siderophores; the production of amino acids and vitamins; the synthesis of ACC deaminase that modulates the level of plant hormones; the mineralization of organic phosphate and the solubilization of inorganic phosphate, which improve the availability of phosphorus for the plant; and the synthesis of growth regulators such as cytokinins, gibberellic acid, and indole acetic acid [9,37]. Additionally, *B. subtilis* can indirectly promote plant growth through indirect mechanisms, including the synthesis of antibiotics, hydrogen cyanide (HCN), and volatile compounds that induce acquired systemic resistance in the plants [37]. *Bacillus methylotrophicus* DD-1 possesses the ability to produce gibberellic acid (GA), indole-3-acetic acid (IAA), and siderophores, which can increase root and shoot length and the dry weight of rice seedlings [42]. The ability of microorganisms to improve plant growth involves several mechanisms such as phosphorus solubilization, nitrogen fixation, and the production of phytohormones [10,42]. It was reported that *B. cereus* YN917 can enhance seed germination and seedling growth through the production of ACC deaminases, siderophores, indole-3-acetic acid (IAA), phosphate solubilizing, and enzymes such as cellulase, amylase, protease, and β-1,3-glucanase [10]. Overall, the mechanism is diverse and complex, which should be studied further. *B. subtilis* may have a significant impact on the intricate plant–environment–pathogen system through biofilm formation, plant growth promotion, competition for resources or colonization sites, cell lysis effects, induced systemic resistance (ISR), and antibiotic production [43]. On the other hand, the increases in antioxidant capacity, total phenolic content, and flavonoids could be due to the plant growth promotion properties of *Bacillus* strains and the biotic stress that was resulted by the phytopathogens. Consequently, this can enhance the antioxidants and/or phytohormones biosynthesis that can mitigate the oxidative stress in seedlings of cotton plants. The phenolic and flavonoid compounds possessed antioxidant and antimicrobial properties and can support plants against the infection of phytopathogens when accumulated in plant tissues [16]. Higher phenolic content in plants can scavenge free radicals and protect the infected plants through cell wall formation and defense mechanisms. In addition to their antimicrobial properties, phenols are structural polymer precursors such as lignin, and can serve as signal molecules to express defense-linked genes [15]. In this context, the total phenolic and flavonoids were increased up to 2.3- and 1.9-fold in tomato plants inoculated with both *B. subtilis* IAGS174 + *Fusarium*, and 1.4- and 1.3-fold in plants inoculated with *Fusarium* alone, respectively, compared to untreated plants [16]. Finally, according to our results, the three *B. subtilis* strains (LDA-1, LDA-2, and LDA-3) have biotechnological potential in the biocontrol of fungal pathogens and plant growth promotion in cotton plants.

## 5. Conclusions

The three types of isolated and identified *B. subtilis* bacteria from the rhizosphere of *P. glandulosa* exhibited significant antifungal activity against the five *Fusarium* species (*Fusarium nygamai*, *F. equisseti*, *F. solani*, *F. solani* ICADL1, and *F. oxysporum* ICADL2). In the pot trials, the strains of *B. subtilis* were able to protect cotton seedlings from the phytopathogenic fungus of *Fusarium solani* ICADL1 as a result of their plant growth-promoting properties such as IAA and GA_3_ production. The synthesis of antimicrobial peptides was linked to the antifungal activity of the bacterial strains, where the presence of genes encoding for lipopeptide (subtilosin, subtilisin, and iturin) production were linked to considerable antifungal activity. As a result, *B. subtilis* strains LDA-1, LDA-2, and LDA-3 could be used as a bioinoculant for root rot disease management and to enhance plant growth of strategic crops such as cotton. However, further investigation is required to demonstrate the biocontrol capabilities of those strains on the large scale of cotton fields grown under stress in the natural conditions.

## Figures and Tables

**Table 1:** Primers used in the present study.

Primer	Sequence	Amplification (bp)	Gene	Reference
27F	(5’- AGAGTTTGATCCTGGCTCAG-3’)	1400	16S rDNA	[22]
1495R	(5’- CTACGGCTACCTTGTTACGA-3’)			
ITS4	(5’-TCCTCCGCTTATTGATATGC-3’)	650	ITS Region	[23]
ITS5	(5’-GGAAGTAAAAGTCGTAACAAGG-3’)			
Qk1F	(5’-CTTAAACGTCAGAGGCGGAG-3’)	704	Subtilisin	[24]
Qk1R	(5’-ATTGTGCAGCTGCTTGTACG-3’)			
Sbo1F	(5’-TCGGTTTGTAAACTTCAACTGC-3’)	334	Subtilosin	
Sbo1R	(5’-GTCCACTAGACAAGCGGCTC-3’)			
ituA1F	(5’-TGCCAGACAGTATGAGGCAG-3’)	885	Iturin	
ituA1R	(5’-CATGCCGTATCCACTGTGAC-3’)			

**Table 2:** Treatments and their abbreviations.

Number	Treatment Name	Abbreviation of Treatment Name
1	Control (distilled water)	C
2	*B. subtilis* LDA-1	LDA-1
3	*B. subtilis* LDA-2	LDA-2
4	*B. subtilis* LDA-3	LDA-3
5	LDA-1+ LDA-2+ LDA-3	Mix
6	Control + *F. solani* ICADL1	C + F
7	*B. subtilis* LDA-1 + *F. solani* ICADL1	LDA-1 + F
8	*B. subtilis* LDA-2 + *F. solani* ICADL1	LDA-2 + F
9	*B. subtilis* LDA-3 + *F. solani* ICADL1	LDA-3 + F
10	LDA-1+ LDA-2+ LDA-3 + *F. solani* ICADL1	Mix + F

**Table 3:** Values of length and maximum and minimum heights of the fungal cell surface of *F. solani* ICADL1 observed by AFM.

Treatments	Length	Height	
		Maximum	Minimum
Control	18.5 ± 0.7 ^a^	1156 ± 25 ^c^	822 s± 16 ^b^
LDA-1	16.0 ± 1.1 ^a^	2032 ± 49 ^a^	653 ± 4 ^c^
LDA-2	16.3 ± 1.0 ^a^	1514 ± 58 ^b^	913 ± 34 ^a^
LDA-3	11.5 ± 0.7 ^b^	1236 ± 57 ^c^	211 ± 3 ^d^
Significance	*	**	***

Values with different letters in the same column are significantly different (*p* < 0.05). The data are presented as means of three replicates, ± standard error (SE). * = *p* ≤ 0.05, ** = *p* ≤ 0.01, *** = *p* ≤ 0.001

**Table 4:** Effect of *B. subtilis* strains (LDA-1, LDA-2, and LDA-3) on physiological parameters and phytohormones (IAA and GA3) in cotton seedlings exposed to *F. solani* ICADL1.

Treatments	IAA (µg/g)	GA (µg/g)	Root Length (cm)	Stem Length (cm)	N. of Secondary Roots
Control	5.4 ± 0.06 ^e^	1.3 ± 0.0005 ^fg^	22.0 ± 1.0 ^c^	8.7 ± 0.3 ^cd^	15.0 ± 0.6 ^e^
LDA-1	9.8 ± 0.2 ^b^	1.1 ± 0.0005 ^h^	14.3 ± 0.3 ^d^	7.7 ± 0.3 ^d^	21.3 ± 0.7 ^d^
LDA-2	6.1 ± 0.24 ^de^	2.5±0.03 ^a^	25.3 ± 0.6 ^b^	13.0 ± 0.6 ^a^	25.0 ± 0.6 ^c^
LDA-3	14.7 ± 0.14 ^a^	1.7±0.03 ^d^	28.7 ± 0.9 ^a^	9.0 ± 0.6 ^cd^	38.7 ± 0.7 ^a^
Mix	6.9 ± 0.18 ^cd^	0.8 ± 0.0005 ^i^	23.3 ± 0.9 ^bc^	11.3 ± 0.7 ^abc^	29.7 ± 0.3 ^b^
Control + F	5.4 ± 0.09 ^e^	1.4 ± 0.0005 ^f^	8.3 ± 0.3 ^f^	9.3 ± 0.3 ^bcd^	20.0 ± 0.6 ^d^
LDA-1 + F	6.1 ± 0.07 ^de^	1.2 ± 0.03 ^g^	11.7 ± 0.3 ^de^	12.0 ± 1.1 ^ab^	8.3 ± 0.3 ^f^
LDA-2 + F	7.4 ± 0.3 ^c^	1.6 ± 0.02 ^e^	9.0 ± 0.6 ^ef^	9.0 ± 0.6 ^cd^	25.7 ± 0.9 ^c^
LDA-3 + F	6.6 ± 0.03 ^cd^	2.1 ± 0.0005 ^b^	9.7 ± 0.3 ^ef^	8.7 ± 0.3 ^cd^	26.3 ± 0.9 ^bc^
Mix + F	5.4 ± 0.44 ^e^	1.9 ± 0.0005 ^c^	11.3 ± 0.3 ^def^	11.0 ± 0.6 ^abc^	29.7 ± 0.9 ^b^
Significance	***	***	***	***	***

Values having different letters, in the same column, are significantly different according to Tukey’s test (*p* < 0.05). The data are presented as means of three replicates, ± standard error (SE). *** = *p* ≤ 0.001

## Data Availability

Not applicable.

## Supplementary Materials

The following supporting information can be downloaded at: https://www.mdpi.com/article/10.3390/biology12010073/s1, Figure S1: PCR amplification of Lipopeptides (subtilosin, subtilisin and iturin) genes from B. subtilis strains (LDA-1, LDA-2 and LDA-3).

## References

[1] Man M., Zhu Y., Liu L., Luo L., Han X., Qiu L., Li F., Ren M., Xing Y. (2022). Defense Mechanisms of Cotton *Fusarium* and *Verticillium* Wilt and Comparison of Pathogenic Response in Cotton and Humans. Int. J. Mol. Sci..

[2] De Farias O.R., De Nascimento L.C., Lima Cruz J.M.F.d., Oliveira Silva H.A., Oliveira M.D.d.M., Alcântara Bruno R.d.L., Castro Arriel N.H. (2019). Biocontrol Potential of *Trichoderma* and *Bacillus* Species on *Fusarium oxysporum* f. sp. *vasinfectum*. J. Exp. Agric. Int..

[3] Abdelmoteleb A., Gonzalez-Mendoza D., Valdez-Salas B., Grimaldo-Juarez O., Ceceña-Duran C. (2018). Inhibition of *Fusarium solani* in Transgenic Insect-Resistant Cotton Plants Treated with Silver Nanoparticles from *Prosopis glandulosa* and *Pluchea sericea*. Egypt. J. Biol. Pest Control.

[4] Abd-Elsalam K.A., Omar M.R., El-Samawaty A.R., Aly A.A. (2007). Response of Commercial Cotton Cultivars to *Fusarium solani*. Plant Pathol. J..

[5] Khan N., Martínez-Hidalgo P., Ice T.A., Maymon M., Humm E.A., Nejat N., Sanders E.R., Kaplan D., Hirsch A.M. (2018). Antifungal Activity of *Bacillus* Species against *Fusarium* and Analysis of the Potential Mechanisms Used in Biocontrol. Front. Microbiol..

[6] Hewedy O.A., Abdel Lateif K.S., Seleiman M.F., Shami A., Albarakaty F.M., M. El-Meihy R. (2020). Phylogenetic Diversity of *Trichoderma* Strains and Their Antagonistic Potential against Soil-Borne Pathogens under Stress Conditions. Biology.

[7] Hong S., Kim T.Y., Won S.-J., Moon J.-H., Ajuna H.B., Kim K.Y., Ahn Y.S. (2022). Control of Fungal Diseases and Fruit Yield Improvement of Strawberry Using *Bacillus velezensis* CE 100. Microorganisms.

[8] Mardanova A.M., Hadieva G.F., Lutfullin M.T., Khilyas I.V., Minnullina L.F., Gilyazeva A.G., Bogomolnaya L.M., Sharipova M.R. (2016). *Bacillus subtilis* Strains with Antifungal Activity against the Phytopathogenic Fungi. Agric. Sci..

[9] Ahmed H.F.A., Seleiman M.F., Al-Saif A.M., Alshiekheid M.A., Battaglia M.L., Taha R.S. (2021). Biological Control of Celery Powdery Mildew Disease Caused by *Erysiphe heraclei* DC In Vitro and In Vivo Conditions. Plants.

[10] Li E., Li Y., Dai X., Yan W., Wang G. (2022). Identification of Two *Bacillus* Strains with Antimicrobial Activity and Preliminary Evaluation of Their Biocontrol Efficiency. Horticulturae.

[11] Saravanan R., Nakkeeran S., Saranya N., Senthilraja C., Renukadevi P., Krishnamoorthy A.S., El Enshasy H.A., El-Adawi H., Malathi V.G., Salmen S.H. (2021). Mining the Genome of *Bacillus velezensis* VB7 (CP047587) for MAMP Genes and Non-Ribosomal Peptide Synthetase Gene Clusters Conferring Antiviral and Antifungal Activity. Microorganisms.

[12] Chen L., Heng J., Qin S., Bian K. (2018). A Comprehensive Understanding of the Biocontrol Potential of *Bacillus velezensis* LM2303 against *Fusarium* Head Blight. PLoS ONE.

[13] Jimenez-Quiros C., Okechukwu E.C., Hong Y., Baysal Ö., Tör M. (2022). Comparison of Antifungal Activity of *Bacillus* Strains against *Fusarium graminearum* In Vitro and In Planta. Plants.

[14] Hashem A., Abd_Allah E.F., Alqarawi A.A., Radhakrishnan R., Kumar A. (2017). Plant Defense Approach of *Bacillus subtilis* (BERA 71) against *Macrophomina phaseolina* (Tassi) Goid in Mung Bean. J. Plant Interact..

[15] Jamil A. (2021). Antifungal and Plant Growth Promoting Activity of *Trichoderma* Spp. against *Fusarium oxysporum* f. sp. *lycopersici* Colonizing Tomato. J. Plant Prot. Res..

[16] Akram W., Ahmad A., Yasin N.A., Anjum T., Ali B., Fatima S., Ahmed S., Simirgiotis M.J., Li G. (2021). Mechanical Strengthening and Metabolic Re-Modulations Are Involved in Protection against *Fusarium* Wilt of Tomato by *B. subtilis* IAGS174. J. Plant Interact..

[17] Abdelmoteleb A., Troncoso-Rojas R., Gonzalez-Soto T., González-Mendoza D. (2017). Antifungical Activity of Autochthonous *Bacillus subtilis* Isolated from *Prosopis juliflora* against Phytopathogenic Fungi. Mycobiology.

[18] Gohil R.B., Raval V.H., Panchal R.R., Rajput K.N. (2022). Plant Growth-Promoting Activity of *Bacillus* Sp. PG-8 Isolated From Fermented Panchagavya and Its Effect on the Growth of *Arachis hypogea*. Front. Agron..

[19] Yamamoto S., Shiraishi S., Suzuki S. (2015). Are Cyclic Lipopeptides Produced by *Bacillus amyloliquefaciens* S13-3 Responsible for the Plant Defence Response in Strawberry against *Colletotrichum gloeosporioides*?. Lett. Appl. Microbiol..

[20] Abdelmoteleb A., Valdez-Salas B., Ceceña-Duran C., Tzintzun-Camacho O., Gutiérrez-Miceli F., Grimaldo-Juarez O., González-Mendoza D. (2017). Silver Nanoparticles from *Prosopis glandulosa* and Their Potential Application as Biocontrol of *Acinetobacter calcoaceticus* and *Bacillus cereus*. Chem. Speciat. Bioavailab..

[21] Mendez-Trujillo V., Moreno-Ramírez L., Carrillo-Beltran M., González-Mendoza D. (2013). Fast Protocol for DNA Isolation of DNA from Bacterial Isolated from a Hyper-Arid Environment. J. Pure Appl. Microbiol..

[22] Zhang A.-M., Zhao G.-Y., Gao T.-G., Wang W., Li J., Zhang S.-F., Zhu B.-C. (2013). Solubilization of Insoluble Potassium and Phosphate by *Paenibacillus kribensis* CX-7: A Soil Microorganism with Biological Control Potential. Afr. J. Microbiol. Res..

[23] White T.J., Bruns T., Lee S., Taylor J. (1990). Amplification and Direct Sequencing of Fungal Ribosomal RNA Genes for Phylogenetics. PCR Protoc..

[24] Cao Y., Xu Z., Ling N., Yuan Y., Yang X., Chen L., Shen B., Shen Q. (2012). Isolation and Identification of Lipopeptides Produced by *B. subtilis* SQR 9 for Suppressing *Fusarium* Wilt of Cucumber. Sci. Hortic..

[25] González-Mendoza D., Argumedo-Delira R., Morales-Trejo A., Pulido-Herrera A., Cervantes-Díaz L., Grimaldo-Juarez O., Alarcón A. (2010). A Rapid Method for Isolation of Total DNA from Pathogenic Filamentous Plant Fungi. Genet. Mol. Res..

[26] Bessai A.S., Bensidhoum L., Nabti E. (2022). Optimization of IAA Production by Telluric Bacteria Isolated from Northern Algeria. Biocatal. Agric. Biotechnol..

[27] Abdelmoteleb A., González-Mendoza D. (2020). A Novel *Streptomyces* Rhizobacteria from Desert Soil with Diverse Anti-Fungal Properties. Rhizosphere.

[28] Abdelmoteleb A., Troncoso-Rojas R., Tzintzun-Camacho O., González-Mendoza D., Duran C.C., Grimaldo-Juárez O., Aviles-Marin M., Duran-Hernández D. (2017). Biocontrol of *Fusarium* spp., Causal Agents of Damping-off in Cotton Plants by Native *Bacillus subtilis* Isolated from *Prosopis juliflora*. Int. J. Agric. Biol..

[29] Ben Slimene I., Tabbene O., Djebali N., Cosette P., Schmitter J.M., Jouenne T., Urdaci M.C., Limam F. (2012). Putative Use of a *Bacillus subtilis* L194 Strain for Biocontrol of *Phoma medicaginis* in *Medicago truncatula* Seedlings. Res. Microbiol..

[30] Aryal S., Baniya M.K., Danekhu K., Kunwar P., Gurung R., Koirala N. (2019). Total Phenolic Content, Flavonoid Content and Antioxidant Potential of Wild Vegetables from Western Nepal. Plants.

[31] Ruiz-Cisneros M.F., Ornelas-Paz J.d.J., Olivas-Orozco G.I., Acosta-Muñiz C.H., Salas-Marina M.Á., Molina-Corral F.J., Berlanga-Reyes D.I., Fernández-Pavía S.P., Cambero-Campos O.J., Rios-Velasco C. (2022). Effect of Rhizosphere Inoculation with *Bacillus* Strains and Phytopathogens on the Contents of Volatiles and Human Health-Related Compounds in Tomato Fruits. Food Sci. Technol..

[32] Ezrari S., Mhidra O., Radouane N., Tahiri A., Polizzi G., Lazraq A., Lahlali R. (2021). Potential Role of Rhizobacteria Isolated from Citrus Rhizosphere for Biological Control of Citrus Dry Root Rot. Plants.

[33] Ntushelo K., Ledwaba L.K., Rauwane M.E., Adebo O.A., Njobeh P.B. (2019). The Mode of Action of *Bacillus* Species against *Fusarium graminearum*, Tools for Investigation, and Future Prospects. Toxins.

[34] Zhao M., Liu D., Liang Z., Huang K., Wu X. (2022). Antagonistic Activity of *Bacillus subtilis* CW14 and Its β-Glucanase against *Aspergillus ochraceus*. Food Control.

[35] Perez K.J., Viana J.d.S., Lopes F.C., Pereira J.Q., dos Santos D.M., Oliveira J.S., Velho R.V., Crispim S.M., Nicoli J.R., Brandelli A. (2017). *Bacillus* spp. Isolated from Puba as a Source of Biosurfactants and Antimicrobial Lipopeptides. Front. Microbiol..

[36] Mora I., Cabrefiga J., Montesinos E. (2015). Cyclic Lipopeptide Biosynthetic Genes and Products, and Inhibitory Activity of Plant-Associated Bacillus against Phytopathogenic Bacteria. PLoS ONE.

[37] Sagar A., Yadav S.S., Sayyed R.Z., Sharma S., Ramteke P.W., Islam M.T., Rahman M., Pandey P. (2022). *Bacillus subtilis*: A Multifarious Plant Growth Promoter, Biocontrol Agent, and Bioalleviator of Abiotic Stress. Bacilli in Agrobiotechnology: Plant Stress Tolerance, Bioremediation, and Bioprospecting.

[38] Goswami M., Deka S. (2020). Isolation of a Novel Rhizobacteria Having Multiple Plant Growth Promoting Traits and Antifungal Activity against Certain Phytopathogens. Microbiol. Res..

[39] Wang C., Zhao D., Qi G., Mao Z., Hu X., Du B., Liu K., Ding Y. (2019). Effects of *Bacillus velezensis* FKM10 for Promoting the Growth of *Malus hupehensis* Rehd. and Inhibiting *Fusarium verticillioides*. Front. Microbiol..

[40] Saleemi M., Kiani M.Z., Sultan T., Khalid A., Mahmood S. (2017). Integrated Effect of Plant Growth-Promoting Rhizobacteria and Phosphate-Solubilizing Microorganisms on Growth of Wheat (*Triticum aestivum* L.) under Rainfed Condition. Agric. Food Secur..

[41] Wang X., Xie H., Ku Y., Yang X., Chen Y., Yang N., Mei X., Cao C. (2020). Chemotaxis of *Bacillus cereus* YL6 and Its Colonization of Chinese Cabbage Seedlings. Plant Soil.

[42] Liu Z., Wang H., Xu W., Wang Z. (2020). Isolation and Evaluation of the Plant Growth Promoting Rhizobacterium *Bacillus methylotrophicus* (DD-1) for Growth Enhancement of Rice Seedling. Arch. Microbiol..

[43] Wang X.Q., Zhao D.L., Shen L.L., Jing C.L., Zhang C.S., Meena V.S. (2018). Application and Mechanisms of *Bacillus subtilis* in Biological Control of Plant Disease. Role of Rhizospheric Microbes in Soil: Volume 1: Stress Management and Agricultural Sustainability.

