# Supplementary material for: New Bacillus subtilis Strains Isolated from Prosopis glandulosa Rhizosphere for Suppressing Fusarium Spp. and Enhancing Growth of Gossypium hirsutum L
Source: Biology (Basel). 2022 Dec 31;12(1):73. doi: 10.3390/biology12010073 (PMC9855134; doi:10.3390/biology12010073)
Supplement: Supplementary file 1 [file biology-12-00073-s001.zip › biology-2118872-supplementary.pdf]

Supplementary

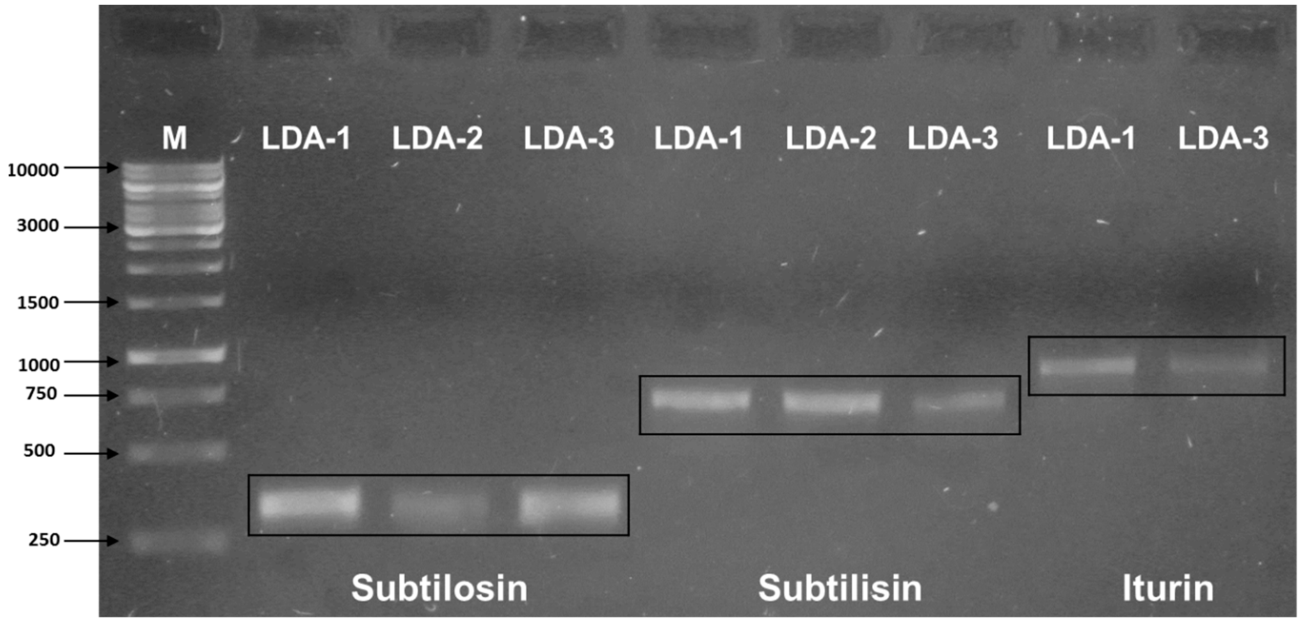

Supplementary Figure S1: PCR amplification of Lipopeptides (subtilosin, subtilisin and iturin) genes from *B. subtilis* strains (LDA-1, LDA-2 and LDA-3).
